# How to transform urban institutional green spaces into Ancillary Botanic Gardens to expand informal botanical learning opportunities in cities

**DOI:** 10.1038/s41598-023-41398-6

**Published:** 2023-09-20

**Authors:** M. Melhem, A. Forrest, Y. Abunnasr, R. Abi Ali, S. N. Talhouk

**Affiliations:** 1 Department of Landscape Design and Ecosystem Management, Faculty of Agricultural and Food Sciences, American University of Beirut, Beirut, Lebanon; 2 Centre for Middle Eastern Plants, Royal Botanic Garden Edinburgh, Edinburgh, Scotland, UK; 3 AUB (American University of Beirut) Botanic Garden (AUBotanic), American University of Beirut, Beirut, Lebanon

**Keywords:** Environmental sciences, Environmental social sciences, Community ecology, Ecosystem services, Urban ecology

## Abstract

Since many cities lack botanical gardens, we introduced the concept of Ancillary Botanic Gardens (ABG), which builds on the premise that organizations can expand informal botanical learning by adding a secondary function to their institutional green spaces. This study guides the application of the ABG concept in various spatial and functional contexts by offering practical and interpretive tools to organizations who are less used to working with nature but are interested in mitigating urban residents’ detachment from nature. Online maps of 220 botanic gardens were reviewed to define types of plant collections and produce an exhaustive list of physical botanic garden elements. The collected information was developed into an ABG field checklist that was tested on three case studies in Lebanon and then used to develop guidelines for ABG establishment. The guidelines and checklist are meant to empower and guide organizations interested in establishing an ABG.

## Introduction

In cities, absence of nature from everyday life leads to plant ‘blindness’, a ‘defective world view’, and contributes to detachment from, and destruction of, the natural world^1–8^.

Urban green spaces offer possibilities for residents to connect with nature especially in cities where there are limited possibilities to interact with the natural world. This opportunity to informally learn about plants and nature beyond schools and universities is described as “learner-motivated, guided by learner interests, voluntary, personal, and open-ended"^9,10^. However, exposure to plants in urban green spaces does not necessarily lead to an educational encounter. This is because many people are plant blind i.e., they tend to be unaware of plants and perceive them as lifeless, static, classified as ‘bulk categories’^11^, lost in the ‘chromatic homogeneity’, and harmless^6^. Plants are the ‘least understood and botanical learning is on the decline leading to an increasing gap in botanical knowledge and patchy and inconsistent conservation activities around the world^2,12^.

Botanic gardens are a type of green spaces that cater for informal botanical learning and for exchange between visitors and plants, evoking a sense of relationship to ecosystems and offering opportunities to explore complex questions about humans and their impact and relationship to other species^13^. When considering typologies of urban green spaces, botanic gardens fall at the center of a continuum, mediating between human experience normally offered by public parks and scientific understanding typically occurring is ecological restoration sites^14^.

However, the geographical distribution of botanic gardens in the world is skewed, and their contribution to informal botanical learning is limited globally. The number of botanic gardens is highest in Europe while most biodiverse areas, such as the tropics, have a small number of botanic gardens and countries in desert biomes, such as those of the Arab League, have the lowest number of botanic gardens^15–18^. Heywood^19^ highlighted this imbalance and showed that biodiversity rich countries have few botanic gardens and conservation activities. Westwood et al.^20^ called for a massive scale to create new gardens in biodiversity hotspots and low-income economy countries where conservation priority is the greatest.

Physical and financial limitations limit the possibility of a widespread increase in the number botanic gardens globally to mitigate plant blindness and mainstream conservation activities. Although there are several sources to guide the establishment of new botanic gardens or the upgrade of existing ones^21–25^, it is unlikely that countries struggling economically will dedicate land and resources in the city to set up and manage botanic gardens^17^. In fact, there is evidence that the persistence of current botanic gardens may be at stake. There are cases where urban development projects have taken over gardens in Kashmir and Iran, with Kashmir losing around 50% of its garden cover in the last decades^26,27^.

Transforming institutional green spaces in cities into botanic gardens is a solution proposed by Talhouk et al.^17^ to increase venues that offer informal botanical learning opportunities. The authors termed these Ancillary Botanic Gardens to emphasize the multifunctional use of urban green spaces, most of which were initially designed for aesthetic purposes. Talhouk et al.^17^ explained that, reconceiving urban green spaces physically and operationally as botanic gardens could offer similar educational roles. ABGs can “develop appropriate attitudes and behavior that may ultimately be responsible for saving the earth”^28–31^, allow visitors to “reflect of their evolving relationship with plants and the rest of the natural world”, and serve as places that will “continue to remind us of the many wonders of life here on earth”^32,33^. Talhouk et al.^17^ explained that Ancillary botanic gardens are secondary on a spatial level in that they are developed in green spaces of archaeological sites, public and private institutions, educational institutions, and touristic sites and institutions. A key aspect of ABGs is that unlike botanic gardens, both their role and scope are flexible rather than prescriptive and are not benchmarked against international standards^22^. This, however, should not lead to the conclusion that ABGs are ‘mere’ visits to green spaces because they are implemented following a locally driven mission to offer informal botanical learning.

Like urban botanic gardens, ABGs can offer informal botanical learning encounters and contribute to sustainability, and global conservation^34^. Depending on how and when they were set up, ABGs, like botanic gardens, can offer unique experiences of nature, telling a story about the culture or history of a community or geographic location^35^. They can use their premises holistically to raise awareness of environmental issues and conduct many activities that contribute to the achievement of the SDGs (Sustainable Development Goals)^16^.

This paper examines the basic physical and functional elements of a botanic garden and argues that the application of the concept of Ancillary Botanic Garden is feasible, that when urban green spaces are conceived from this new perspective, they may expand informal botanical learning opportunities in cities. By proposing field assessment tools and universal ABG guidelines the authors propose a novel way to bring out the identity and value of plants in urban green spaces where they were placed as aesthetic backdrops to various institutions. The aim is to offer a road map for public and private institutions who are less used to working with nature to help mitigate urban residents’ detachment from nature. The ABG road map to guide the transformation of urban institutional green spaces into ABGs includes evidence-based tools that consist of guidelines, a field checklist, and a description of botanic garden elements. The tools developed by the study provide a common method, yet they offer interpretive flexibility guiding the transformation of urban institutional green spaces into ABGs in a variety of spatial and functional contexts.

## Methods

**Figure 1:**
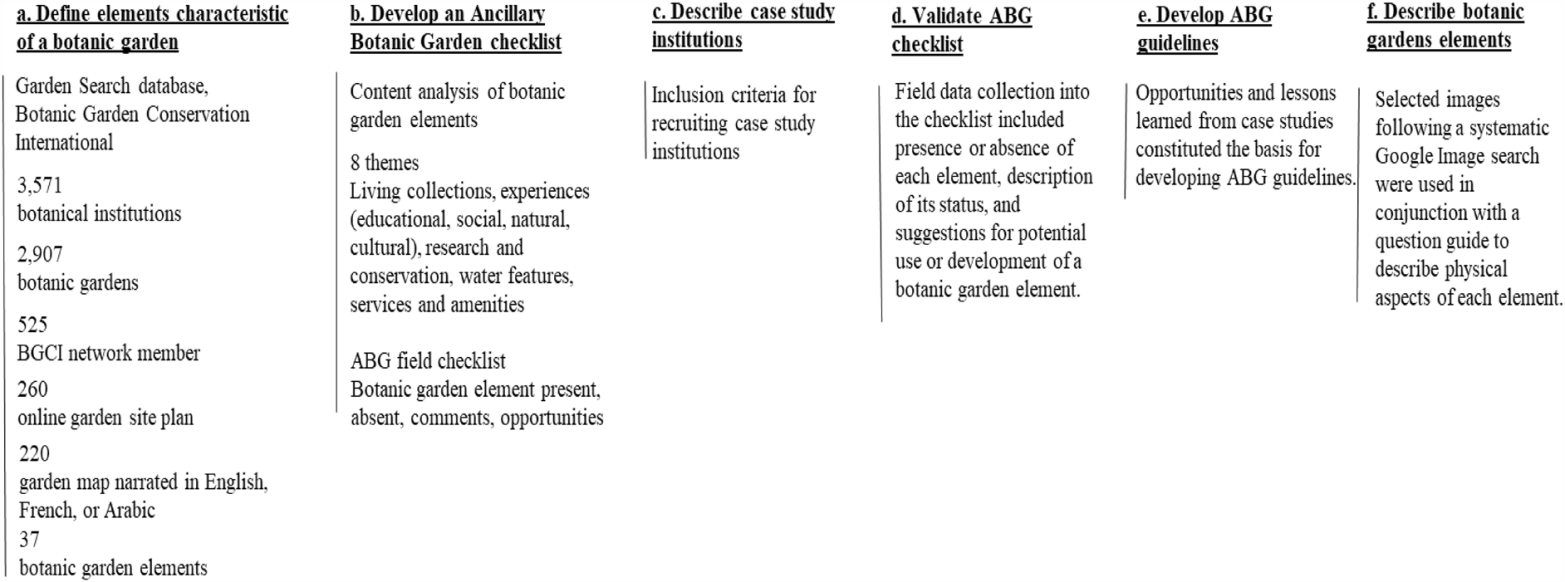
Flowchart depicting the steps followed to identify the botanic garden elements, develop the ABG field checklist, the ABG guidelines, and the description of the botanic garden elements.

The following flowchart depicts the various steps followed to develop the ABG guidelines (Fig. 1):

### Defining elements characteristic of a botanic garden

The Garden Search database hosted by Botanic Garden Conservation International (BGCI), was used as the source for primary data collection and included, at the time the research was conducted, 3571 botanical institutions including botanic gardens, gene/seed banks, zoological institutions, private collections, and networks^36^. Characteristic elements of a botanic garden were found following a systematic analysis of 220 case study botanic gardens featured in the Garden Search global database (https://www.bgci.org/resources/bgci-databases/gardensearch/). Case study institutions included in the study were: botanic gardens (2907) that are members of the BGCI network (525) and have published a garden site plan online (260), which is narrated in English, French, or Arabic (220).

Data collection consisted of systematically inspecting the 220 botanic garden site plans and recording all marked elements in a spreadsheet, adding new entries for every newly encountered element. This iterative process of inspecting botanic garden maps and adding new entry fields to the spreadsheet continued until no new elements were found, and all botanic gardens with their elements shown on maps were accounted for (M. Melhem, and S.N. Talhouk conducted the content analysis and organized the elements into thematic categories without pre-existing frameworks).

### Developing an Ancillary Botanic Garden checklist

The categorized botanic garden elements were organized into a matrix referred to as ABG field checklist and featuring columns that allow for comments on whether the element is present, absent, and comments on potential development of an element (matrix sheet not shown). The ABG field checklist was used for site analysis of cases study institutions.

### Description of case study institutions

The case study institutions were selected based on the following inclusion criteria: (1) the institution has a green space equal to or larger than the built area, (2) the owner is managing the green space, (3) the owner has expressed interest in exploring the possibility of retrofitting the green space into an ABG.

- Case study 1 site primary function is the production of certified organic authentic Lebanese and Mediterranean juices, jams, and preserves. Located northeast of Beirut, the site building, land and old Mediterranean terraces were rehabilitated by the owner who is looking to reconstruct stories and traditions in memory of his grandfather.
- Case study 2 is a residence in Beirut built in 1860. The estate is amongst the largest private homes in the city with a primary function to cater for weddings and exhibitions. The estate suffered extensive damage in the Beirut Blast in August 2020^37^.
- Case study 3 is a private school founded in 1873. The school is east of Beirut and its campus includes a wooded hillside with panoramic views.

### Validating the Ancillary Botanic Garden checklist

Site visits were conducted by researchers to collect data using the ABG checklist in collaboration with the owner or assigned contact person who was present during the visits. The information compiled in the checklist included presence or absence of each element, description of its status, and suggestions for potential use or development of a botanic garden element.

### Developing guidelines for retrofitting urban institutional green spaces into Ancillary Botanic Gardens

Opportunities and lessons learned from the three case studies constituted the basis for developing ABG guidelines.

### Describing functionality, materiality, and modes of use of the recorded botanic gardens elements

To better guide and inform ABG stakeholders, each recorded botanic garden element was described as shown below:

A systematic Google Image search was conducted using the element as key word associated with the word ‘botanic garden’. When the search results produced unrelated images, a new search was performed using the element as the only key word. For each element, the top 20 image hits were examined, and the first five images that clearly showed physical aspects of the element, namely, material it is made of, its relative size, its context location, circulation related to it, types of activities within it, and accessibility to it, were selected. With the selected images, the following set of questions was addressed to complete the image guided description of each botanic garden element: What are the materials (soft scape, hardscape) used to construct the element? What is the estimated size of the element? Where is the element found? How is the circulation organized within or around the element? What do people do in the element? What is the main program of activities in the element? How is the element accessible? Is there any remarkable observation which was not included in the answers above?

## Results

### General description of case study botanic gardens

**Figure 2:**
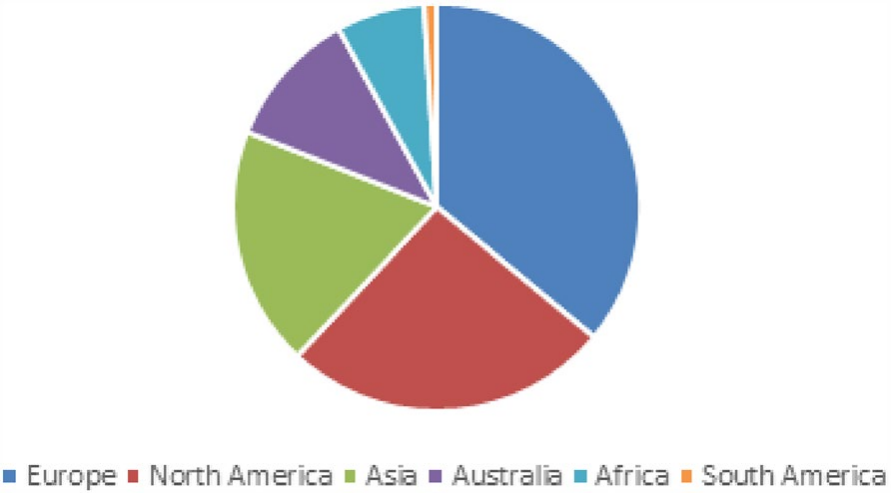
Geographic distribution by percentage of case study botanic gardens.

The case study botanic gardens (N = 220) are in Europe (36%), North America (26%), Asia (19%), Australia (11%), Africa (7%), and South America (1%) (Fig. 2). More than half (64%) of these botanic gardens are public, 20% are privately owned, and 16% are owned by academic/educational institutions.

The establishment dates of the case study botanic gardens span over 470 years. Using the five historical periods that coincide with changing roles of botanic gardens^13,38^, the results show that most case study botanic gardens were set up during the last two periods, i.e., between 1851 and 1960 and from 1960 till today (Table 1).

**Table 1:** Founding date period and role of case study botanic gardens at establishment (N = 220).

Founding period	Botanic garden role^38,39^	Number of botanic gardens
1550–1680	European medicinal	14
1681–1780	Classic European	5
1781–1850	Colonial tropical	26
1851–1960	Civic and municipal/Specialized	91
1961–present	Education/conservation/research/recreation	84

### Typology of plant collections in case study botanic gardens

According to BGCI^36^, plant collections are organized as geographical—consisting of native plant collections from the surrounding region or national flora, taxonomic—displaying taxonomic plant groups, thematic—focusing on related or morphologically similar plants such as orchids and roses, or plants falling under the same theme such as medicinal plants, bonsai, and butterfly gardens, and ecological—including plants species that occur in similar habitats or ecotypes such as alpines or epiphytes^36^.

The number of recorded plant collections in the case study botanic gardens was 350, with some housing more than one collection, and less than half (45%) housing an arboretum. Based on the names assigned to the plant collections, it appears that half were thematic, one third were geographic, and less than 10% were ecological or taxonomic. Further inspection of the thematic plant collections names revealed a diversity of educational intents including habitats (ex: rock garden, moss garden, water garden), horticulture (ex: rose garden, vegetable, xeriscape), culture (ex: thirteenth century, Viking, Shakespeare, Memories), as well as leisure (ex: winter, shade, gravel, entry, exhibition) (Table 2).It is worth noting that the assignment of the collections under the four categories was based on the authors’ assessment of the name of the collection. There may have been overlaps between diverse types of collections that were overlooked such as geographical attributes which are also closely related with ecological and habitat attributes. This may affect the actual number of collections in each category but the fact that the largest numbers were those of thematic and geographic plant collections remains.

**Table 2:** Types and names of plant collections recorded in case study botanic gardens (N = 220, some botanic gardens house more than one collection).

Type of plant collection	Number of plant collections	Examples of names of plant collections
Geographical	110	Eastern Asian, Eurasian bog and heath, American bog and heath, Mexico, Arican, Australian, Bavarian, Californian, Canary Island, Chilean, Chinese, German, Gondwana, Japanese, Madagascar, Mediterranean, New Zealand, Ogasawara islands, SriLankan, South African
Ecological	18	Aquatic, Alpine, Epiphytes, Wetland ecosystem, Dune ecosystem
Taxonomic	13	Bambusetum, Fabaceae, Palmae, Pinetum, Bromeliad
Thematic	190	HABITAT: Rock, Moss, Pond, Desert, Aquatic, Water, Waterfall, Sun, Woodland, Butterfly, Evolution, Conservation
		HORTICULTURE: Woody plants, Annual, Ornamental, Rose, Bamboo, Cut flower, Fern, Succulent, Camellia, Rhododendron, Daylily, Bonsai, Fruits and vegetables, Poisonous plants, Herb, Lilies, Four seasons, Xeriscape
		CULTURE: thirteenth century, Heritage, Viking, Ruin, Historic, Scarecrow, Victorian, Shakespeare, Founder, Teacup, Tea, Memories, Ancient plants, Festival, English walled, Tennis court, Jungle, Cottage, Colonial, Lovers, Enabling, Dinosaurs
		LEISURE: Winter, Vertical, Shade, Floral, Gravel, Entry, Green roof, Exhibition, Welcome, Circle, Courtyard, Fountain, Children

### Botanic garden elements and thematic categories

Thirty-seven botanic garden elements were recorded and organized under seven themes namely, living collections, educational experiences, social experiences, natural experiences, cultural experiences, research and conservation, and water features (Table 3). In addition, there were 12 elements related to services and amenities. The percentage occurrence of each element in the case study botanic gardens was recorded. The highest-ranking elements present in more than 25% of botanic garden maps include plant collections, arboreta, and fauna (living collections), tours-trails and visitor education centers (educational experiences), restaurant-café, gift shop, and pavilion (social experiences), fauna, picnic, and children playground-trail (natural experience), herbarium and seedbank (research and conservation), and pond (water features). The highest-ranking elements under services and amenities included restrooms, parking, information desk, and wheelchair access.

**Table 3:** Category, type, and frequency (%) of botanic garden elements appearing in 220 botanic garden maps.

Living collections	Plant collections (100%), arboreta (45%), Fauna (30%)
Research and conservation	Herbarium (45%), Seed Bank (35%), Nursery (19%), Conservatory/Conservation area (18%), Research center (2%)
Educational experiences	Tours–Trail (40%), Visitor education center (31%), Classroom/educational program/school program (15%), Exhibition (11%), Library (5%), Meeting point (4%), Museum (4%), Signage and Information board (3%), Audio Guide (2%)
Natural experiences	Picnic (26%), Children Playground and trail (24%), Viewing platform (18%), Bike trail (9%), Avenue (5%), Plant sale (4%), Treehouse (1%)
Cultural experiences	Sculpture and monuments (13%), Memorial (12%), Historic section (5%)
Water features	Pond (31%), Lake (16%), Fountain (9%), Wetland (9%)
Social experiences	Restaurant–Café (54%), Gift shop (38%), Pavilion (25%), Amphitheater (16%), Wedding (10%), Barbecue (5%)
Services and amenities	Restroom (64%), Parking (53%), Information desk (29%), Wheelchair access (25%), Shelter (23%), Administrative building (21%), Drinking water (19%), Tram-scooter-shuttle bus (15%), Ticket counter (9%), First aid (8%), Parents room (7%)

### Field testing of the Ancillary Botanic Garden checklist

**Figure 3:**
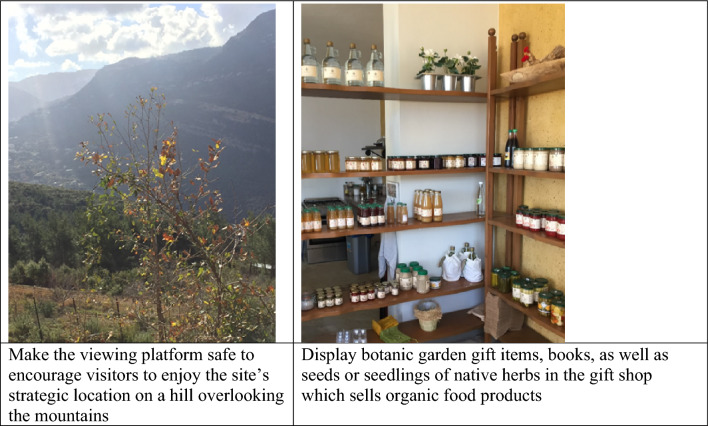
Select images of Case study 1.

**Figure 4:**
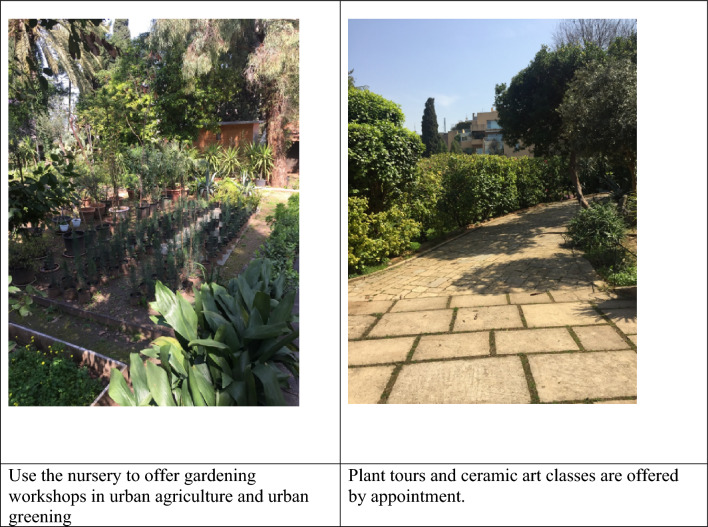
Sample images of case study 2 (The site has been severely damaged by the Beirut Blast (Aug 2021)).

**Figure 5:**
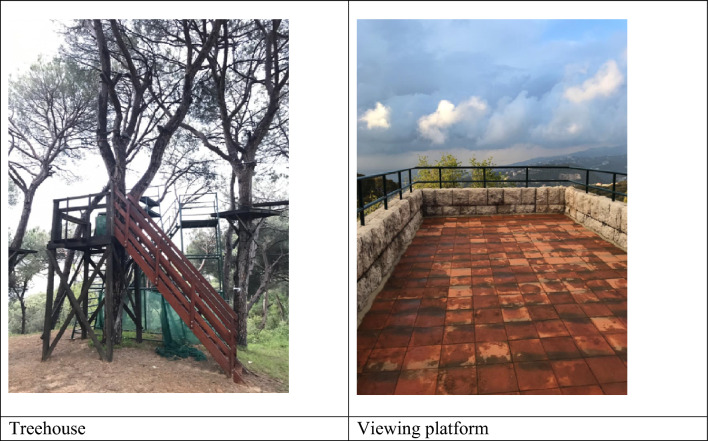
Some representative pictures of case study 3.

For each case study institution, the Ancillary Botanic Garden checklist was completed during field visits as shown in Tables 4, 5, and 6, and Figs. 3, 4, and 5 below.

**Table 4:** Site analysis and recommendations (in italics) for case study 1 using the ABG checklist.

Living collection
Mediterranean native species, wild edible plants, and traditional fruit tree varieties. Sheep and free-range chicken *Create two thematic plant collections 1- Mediterranean terrace habitat 2- traditional horticultural varieties. Label plants with common Arabic names and scientific names. Include information on traditional use of plants. Create a farm animal petting zoo area*
Research and Conservation
Traditional stone terraces planted with aged olive trees *Create a Mediterranean terrace habitat conservation area including the aged olive trees and the associated native flora*
Educational Experiences
The institution does not offer plant tours. It lacks defined plant tour trails, a visitor education space, an exhibition area, a library, and a meeting point. There are limited signage and information boards. Outdoor organic cooking classes are offered for people of all ages including children *Define trails and organize plant tours around the two thematic plant collections. Refurbish one room to offer workshops in the building. Use the existing terrace as a space for exhibition. Dedicate a space in the gift shop for botanical and gardening books. Formalize the historic tree location as a visitor meeting point. Design information signage narrating: 1- native Mediterranean species, 2- wild edible plants, 3- traditional fruit tree varieties. Teach humane treatment of domesticated animals. Explain the importance of traditional terraces in soil and water conservation*
Natural Experiences
The space lacks a picnic area, a children's playground and trail, or a nature-viewing platform. There is no plant sale outlet *Create a natural children free play area and trail. Collect wild edible plant seeds from site and sell herb seeds and seedlings in the gift shop. Ensure safety access to the platform to use the institution’s access to a scenic view of Mediterranean hills and mountains*
Cultural Experiences
Traditional agriculture tools are preserved on site. The owner is passionate about reviving his grandfather's legacy as a traditional farmer. There are ruins of an old mill and wine press *Develop a historical narrative around the owner’s grandfather as a traditional farmer, display and describe how the old mill, wine press, and agricultural tools were used, focusing on nature-based solutions to sustainable agriculture*
Social Experiences
There is a gift shop that sells organic juices, jams, tomato products, and pickles processed from products organically grown by the institution. There is a barbecue grill *Diversify and expand gift shop items to include botanical handcrafts, gift items, seeds and seedlings, and botanical and horticultural books and manuals. Use the grill for offering hands outdoor cooking workshops using wild edible plant-based recipes*
Facilities
There is an administrative building also used as a facility for food processing. There is a sheltered outdoor space that seats 20 people and one bathroom. The space lacks a drinking water source, first aid kits, and an information desk. There is a car park at the entrance but no wheelchair access through the site *Supply clean drinking water through traditional Lebanese drinking jars. Assign location for first aid kit and emergency numbers. Produce informative flyers at reception. Consider a small outdoor private space for parents with infants. Use shelter as an outdoor classroom facility. Explore and define wheelchair accessible areas and activities*

**Table 5:** Site analysis and recommendations (in italics) for case study 2 using the ABG checklist.

Living collection
Trees and shrub species typically used in traditional Beirut gardens. Tortoise and birds *Create a thematic plant collection on trees and shrubs of historic Beirut gardens. Label plants with common Arabic names (if possible) and scientific names. Include information on plant species typically used in traditional Beirut gardens and their natural origin (native or exotic). Introduce elements such as bird feeders, bug hotels, shelters, and water sources to make garden wildlife friendly*
Research and Conservation
The site includes a partially used nursery that supplies the garden's needs, including landscape and edible plants *Develop the nursery set up and operation to engage visitors in gardening workshops*
Educational Experiences
There is no defined trail throughout the estate’s green spaces which is opened to the public, mainly schools and universities, for plant tours by appointment. The school or university instructor typically guides the tour. The owner also offers classes in ceramic art by appointment. Visitors normally meet in the plaza at the estate entrance. There are no plant signs or information boards in the garden *Define a botanic garden visitor trail that goes through the garden. Label plants and add signage narrating the history of the garden. Offer plant tours on historic trees and shrubs of Beirut. Use the nursery to offer gardening workshops in urban agriculture and urban greening. Explain how urban green spaces can become wildlife friendly. Use the outdoor plaza for botanical exhibitions. Define the entrance plaza as a meeting point for visitors*
Natural Experiences
Day use of the space by the public is rare as the garden is primarily meant to accommodate evening events such as receptions and weddings *Explore the possibility of day access to tree shaded garden space as a picnic area for small groups of young children from neighborhood schools. Dedicate part of the nursery for the production and sale of traditional Beiruti garden trees and shrubs*
Cultural Experiences
The owner has an archive of old photos of Beirut and the residence that is open to visitors *The site can serve as a venue to evoke the history of the family along with the history of Beirut. Collaborate with nearby educational institutions to curate the collection of sculptures and monuments dating from 1860*
Social Experiences
The estate has opened its gardens and large plazas wedding events, but it does have a gift shop or coffee shop for day visitors *Direct access to the plaza spaces for outdoor plant talks and discussions on nature-based solutions and urban green spaces. Consider setting up a ‘gift shop’ that sells artisanal or botanical crafts, ceramics prepared at the workshops, and healthy snacks and drinks*
Water features
The site includes a historic fountain *Aquatic plants can be added to diversify plant encounters*
Facilities
An administrative section was refurbished in the estate to accommodate small offices used for booking the wedding venue. A visitor education or information center are not essential since the garden is open to visitors by appointment only and in the presence of a guide. There are public restrooms that are available for wedding guests. The site is not wheelchair accessible, and there is no clear first aid kit location *Expand the function of the offices to book plant tours and other activities in the garden and equip them with first aid kits. Consider which part of the estate is wheelchair accessible*

**Table 6:** Site analysis and recommendations (in italics) for case study 3 using the ABG assessment checklist.

Living collection
Mostly pines and cypresses. The number of species is low. Birds *Thematic plant collection - Mediterranean pine woodland habitat. Introduce understory species adapted to the habitat. Label plants using common and scientific names. Introduce elements such as bird feeders, bug hotels, shelters, and water sources to make a wildlife friendly woodland area*
Research and Conservation
Aged pine and cypress trees grow on traditional stone-built terraces. A plant nursery supports the campus needs for landscape plants *Dedicate a Mediterranean woodland conservation area on campus. Use the nursery to produce and distribute forest seedlings*
Educational Experiences
The institution has defined trails that go through the whole campus. It has many classrooms, large plazas, an outdoor amphitheater, a library, and formal meeting points at the campus gate entrances. Signage is on built structures and for way finding but no information about outdoor spaces *Introduce plant signage and information boards about habitats and plant species on site. Organize plant tours, use classrooms, and indoor amphitheaters to feature educational botanical and nature documentaries. Use plazas for botanical exhibitions. Use outdoor amphitheater for featuring plays and concerts that help support nature conservation. Dedicate a section in the library for botanical and gardening books. Use gate entrances as formal meeting points for visitors*
Natural Experiences
The site includes countless natural experiences; it has tree-lined avenue, treehouses, children’s playgrounds and viewing platforms to the mountains *Dedicate a free play children area, accommodate for a picnic area, and consider a bike trail through the wooded area*
Cultural Experiences
The institution has a memorial for the school founder *Explore the institution’s archive to document the greening of the campus through time. Develop fundraising drives to support the garden through alumni who would adopt a tree or a bench on campus*
Social Experiences
The site has a cafeteria/ restaurant, and a bookshop *Use the cafeteria during botanical events. Dedicate a botanical section for books and gifts in the bookshop*
Water features
The site has a local water source that could be further developed into a water feature
Facilities
The institution is fully equipped to receive visitors. It has an administration building, an education center, parking, restroom, drinking water, and first aid

### Guidelines for retrofitting institutional green spaces to Ancillary Botanic Gardens

This section elaborates general guidelines for Ancillary Botanic Gardens derived from the content analysis, field data observations, reflection about the applicability of the ABG concept following the receptivity of the stakeholders that took part in this exercise, and the authors experience in transforming the campus of the American University of Beirut into an Ancillary Botanic Garden^40^. The ABG guidelines shown below, along with the ABG checklist, and the description of each botanic garden element, are meant to inform and guide institutions interested in setting up an ABG on their premises to give benefit to the primary function of the institution (Table 7).

**Table 7:** ABG guidelines.

Living collections	• Name plant species, including scientific names and plant origin with the support of a botanical, academic, research, or non-governmental institution • Show local names and cultural values of species when relevant by consulting local resource persons • Label plants • Develop a theme for the plant collection • Create botanical, cultural, or institutional stories about plants and habitats • Elaborate an animal friendly encounter with resident fauna or domesticated animals while considering legal and ethical considerations
Educational experiences	• Forge a partnership with nearby experts • Empower interested members of staff and clients of the institution to lead and organize educational activities • Assign a meeting point for visitors • Define plant tour trails • Use plant signage and information boards to allow self-guided visits • Schedule the use of existing plazas and rooms to accommodate botanical exhibitions and nature educational programs • Organize open-air lectures
Social experiences	• Extend the opening hours of available food facilities to coincide with the scheduled visits to the ABG • 2. Add a section dedicated to plant and nature-based items to existing gift shop
Nature experiences	• Develop trail and nature-based free play space and elements for children • Develop healing nature experiences for adults and elderly • Introduce wildlife friendly elements
Cultural experiences	• Organize public displays about the institution's history • Develop and communicate the institution’s commitment to conservation and community engagement on its premises • Organize open-air artistic events to fundraise for the activities of the ABG
Visitor services	• Offer an inclusive environment, show if and which space is accessible for physically disabled, produce a guide showing accessible areas of the ABG

## Discussion

Building on the concept of Ancillary Botanic Garden, this paper explored how reconceiving institutional urban green spaces could support informal botanical learning by emulating the layout and function of a botanic garden.

With over half its case study botanic gardens found in Europe and North America, the research first confirmed the reported imbalance in the geographic distribution of botanic gardens supporting the argument that many cities around the world lack these botanical institutions^19^. Addressing this skewed distribution, Westwood et al.^20^ called for a massive scale effort to create new gardens in biodiversity hotspots and low-income economy countries where conservation priority is the greatest and where prospects for global conservation efforts will be affected by the gap in botanical knowledge^12^. The outcome of this study, which guides public and private institutions on how to establish ABGs, is a response to calls for increasing botanic gardens worldwide. Furthermore, the proposed application of the ABG concept is innovative and sustainable and contributes to an increase in the number of botanic gardens that are locally grown, multifunctional, financially sustainable, and co-created with community^41^.

To better understand the nature of plant collections typically housed in botanic gardens, the study explored the types of plant collections ‘worthy’ of imparting informal botanical learning in the case study botanic gardens. The findings revealed that most plant collections have a geographic or thematic scope rather than an ecological or taxonomic one. This may be because many of these gardens were set up during periods where the focus was on public education and horticulture rather than research^38,39^. This finding is important in that it points to the fact that a scientific underpinning is not necessary to offer informal botanical learning to the public. In fact, many institutions have developed botanical collections within their institutional grounds to cater for the learning of their constituencies. Schools have set up botanical collections to offer students dynamic and novel educational methods, boost students’ consumption of fruits and vegetables, and instill an early determination for environmental conservation^42–44^. Universities display native and traditionally used plant species on their campuses to promote local heritage and culture. Medical institutions have set up therapy and edible plant gardens to promote well-being^45^. Civil society groups transformed public green spaces into botanic gardens to provide communities with a sense of place ownership, to promote wellbeing, and to serve as a link to traditional knowledge^46,47^. In this research, field assessment of the first case study site revealed that it houses two types of plant collections namely; a Mediterranean-terrace habitat plant collection, and traditional horticultural varieties. The second case study site has a collection of trees and shrubs representative of historic Beirut gardens. The third case study includes a Mediterranean pine woodland habitat. Although all three case study institutions selected plant species with no educational goal in sight, today, these plant collections can contribute to local informal botanical learning. The ABG field checklist and guidelines guide the process of defining the theme of the plant collections in each urban green space depending on the predominance of the species present at the time of the ABG field assessment. Considering that, each urban institution set up its urban green space independently and at various times, when many such institutions engage in this transformation of their green spaces into ABGs, they will offer diverse scale, size, and scope for informal botanical learning through their plant collections. They will also ‘casually’ reach out to a larger number of citizens by targeting their respective constituencies.

Unlike the focus of the literature on the design and establishment of new botanic gardens, this study contributes to grassroots action by offering urban institutions, who may wish to contribute to informal botanic learning, tools that aid in assigning a secondary function to green spaces without jeopardizing their institutional primary function. The importance of botanic gardens as providers of education and recreation is well established^48,49^; and has driven the production of many publications to guide the establishment of new botanic gardens or upgrade of existing ones^21–25^. The tools provided in this study are especially relevant in poor countries and in line with Reid and Gable^50^ who suggested that small-scale horticultural oases such as localized community, school, and demonstration gardens can play the same educational role as botanic gardens and can have multiplied impacts that also include building a sense of community underserved areas.

Regarding the type of institutions that can engage in the establishment of ABGs, the study revealed that more than half of the case study botanic gardens are public, 20% privately owned, and 16% owned by academic/educational institutions. Although the findings of the content analysis suggest an important opportunity to reconceive public green spaces into ABGs, the case study locations in this research were drawn from privately owned green spaces. We do not believe that there is a particular situation in Lebanon that makes private gardens / institutions more prone to be transformed into ABG. However, during the research, it was not possible to secure a case study location standing for a public green space due to political conflicts and economic instability, which left public institutions idle and dysfunctional with limited financial and human resources. On the other hand, Talhouk et al.^51^ showed unique opportunities to expand the scope and breadth of botanical learning in Lebanon by reconceiving public green spaces, specifically the peripheries of archeological sites, which are around 350 and vary from three to 25 hectares, as ABGs. Work is in progress to develop one of these sites as an ABG in collaboration with the Lebanese Ministry of Culture. Efforts are also ongoing to explore green space assets of tourism resorts to contribute to informal botanical learning. By establishing ABGs, public and private institutions may develop a new and different relation with their constituencies, residents, staff, or clients, by sharing a physical green space that promotes the supportive role of nature and by encouraging them to revisit the relationship between plants and people and plants and local cultures^13^.

The botanical garden elements shown after a content analysis of the 220-case study were organized into themes and developed into an ABG field checklist used in field assessments. The checklist allowed for a systematic recording and evaluation on site of each element during field visits, deciding whether the element was present or absent. More importantly, systematically going over the checklist gave the opportunity to co-design with the owners the physical and operational opportunities to develop a given botanic garden element and associated activities. The findings and suggestions arising from this exercise are listed in Tables 4, 5, and 6. This approach is important as it guides a systematic assessment that precedes the transformation of the green space into an ABG while observing locality, i.e., a botanic garden that is locally grown, multifunctional, financially sustainable, and co-created with community^41^.

An important contribution of the ABG field checklist was unveiled during field evaluations of the case study sites. The systematic assessment of the presence, absence, or potential development of an element following clear theme related to experiences in botanic gardens built the confidence of stakeholders in the possibility of contributing to informal botanical learning. Stakeholders saw clarity in the assessment and planning of an ABG which was physically and operationally aligned with formal botanic gardens. Field assessments using the ABG field checklist also opened new perspectives for owners of how their green spaces are seen, and used, and how the institution’s history and culture can be part of it. For example, stakeholders of the case study sites were not aware of the value of their plant collections. The field checklist dispelled the misconception amongst stakeholders that plants with educational value are specific to botanic gardens. Maunder^52^ and Robertson^4^ showed that botanic gardens have been judged, or have judged themselves, by the number of species held in the garden; however, the value of plant collections is in their contribution to economic and social development rather than by the number of the species kept as botanical living dead. Furthermore, Rae^53^ pointed out that it is not uncommon that botanic gardens build up their collection first and then afterwards sort out the justification of the existence of their collections.

The ABG guidelines presented in this study are an application of the ABG concept and were informed by insights gained from the content analysis, the development of the ABG checklist and its field application. While content analysis helped list the basic elements and types of operations in a botanic garden, the use of the ABG checklist during field assessments showed which elements are unlikely to be found in institutional urban green spaces. These elements were kept in the field checklist, but not included as core elements in the guidelines. With respect to living collections, the ABG guidelines were also informed by the field visits to case study sites where it was clear that a holistic view of the vegetation, i.e., landscape of the site including the history of the institution may be an inspiration for naming the living collection. The field visits also highlighted the importance of collaboration between stakeholders within the institution and outside the institution to lead and sustain activities in the ABG. Another element clear from the field visit is that the commitment of an institution to setting up an ABG reflects its commitment to conservation and community engagement, and this commitment should be communicated.

The ABG guidelines are universal in scope, beyond Lebanon, and applicable in various spatial and functional contexts. More importantly, the guidelines incorporate key aspects of the ABG concept^17^. Unlike botanic gardens, ABGs are ‘deregulated’ and seek to promote informal botanical learning, their roles and scope are not benchmarked against international standards^22^, and their mandates are flexible rather than prescriptive and are defined by stakeholders. This does not mean that ABGs are designed as ‘mere’ urban green spaces because they are conceived to offer botanical learning hence the importance of the ABG guidelines, the field checklist, and the description of the elements. Based on physical and operational elements of formal botanic gardens, the ABG guidelines capture the essence of the ABG concept as follows: They rely on local nomenclature which is fundamental in developing enthusiasm for plant conservation, they seek to create themes from existing plant assemblages, essential for effective local communication and engagement, they encourage the engagement of taxonomically illiterate members of society as ‘custodians’ of ethnobotanical knowledge, they guide the development of urban green spaces as local ‘nature’ that is healing for adults, a free play space for children, and wildlife friendly for biodiversity, they link the institution’s culture and history with nature and culture, and they encourage the development of inclusive greenspaces.

With the ABG checklist and the description of the botanic garden elements, the guidelines are the ABG concept’s application. They offer practical and interpretive tools to organizations who are less used to working with nature but are interested in mitigating plant blindness and urban residents’ detachment from nature.

## Conclusion

By exploring the application of the Ancillary Botanic Garden concept, this study has contributed to the mitigation of plant blindness by developing guidelines that guide the repurposing of urban green spaces to bring out the educational value of plants. Based on an analysis of formal botanic gardens, the guidelines, along with the field checklist, and description of elements, are also an example of how to promote collaboration between public and private institutions. On a practical level, the developed tools offer benefits to visitors, allowing regular access to urban green spaces and botanical learning opportunities. They also allow institutions to maximize the use of an underutilized asset and formalize their contribution to nature and human wellbeing. Finally, it is worth noting that one of the case study sites registered as a formal botanic garden following this research, showing that ABGs may be a transitioning step by urban institutions who chose to further commit to botanical learning and join the global botanic garden community by becoming a member of formal botanic garden organizations.

## Data Availability

The datasets generated during and/or analysed during the current study are available from the corresponding author on reasonable request.
